# Retraction: Pimecrolimus Cream 1% in the Management of Atopic Dermatitis in Pediatric Patients: A Meta-Analysis

**DOI:** 10.1371/journal.pone.0108566

**Published:** 2014-09-16

**Authors:** The PLOS ONE Editors

After the publication of the article, a group of readers raised concerns about several aspects of the methodology for the meta-analysis and the interpretation of the results (http://www.plosone.org/article/comments/info%3Adoi%2F10.1371%2Fjournal.pone.0093095).

Upon follow-up with the authors and consultation with editorial board members, the following concerns have been identified:

* The interpretation of the reduction in Investigator's Global Assessment (IGA) score is incorrect (Figure 2), a greater reduction in IGA scores indicates a positive event and thus the analysis shows a beneficial effect of the intervention pimecrolimus.

* Figure 3 is incorrectly labelled, the label 'control' should read 'tacrolimus', the interpretation of the results does not take into consideration the direction of the outcome.

* There are concerns about the heterogeneity of the studies analysed in Figure 4, which suggests that the results of the trials should not be combined. The interpretation of the results of Figure 4 is incorrect, lack of flare constitutes a positive outcome.

* There are concerns about the combination of trials that involved placebo controls with those including active comparators, particularly in relation to analyses regarding the assessment of adverse events.

* The meta-analysis combined randomized and non-randomized trials, the analyses were not stratified by study design.

* There are concerns about the use of blinding as a selection criteria for inclusion in the meta-analysis.

* Some aspects of the reporting do not adhere to the requirements outlined in the PRISMA guidelines and about the reproducibility of the search strategy reported.

The editors consider that the concerns above compromise the validity of the conclusions drawn in relation to the efficacy and safety of pimecrolimus cream 1% for the treatment of atopic dermatitis in paediatric patients and as a result retract this publication.
